# A 7-Hydroxy 4-Methylcoumarin Enhances Melanogenesis in B16-F10 Melanoma Cells

**DOI:** 10.3390/molecules28073039

**Published:** 2023-03-29

**Authors:** Taejin Kim, Kwan Bo Kim, Chang-Gu Hyun

**Affiliations:** Jeju Inside Agency and Cosmetic Science Center, Department of Chemistry and Cosmetics, Jeju National University, Jeju 63243, Republic of Korea

**Keywords:** B16-F10, 7-hydroxy 4-methycoumarin, hypopigmentary, melanogenesis, signaling pathway

## Abstract

The objectives of this study were to investigate the melanogenetic potentials of the naturally occurring 7-hydroxy coumarin derivatives 7-hydroxy 5,6-dimethoxycoumarin (7H-5,6DM), 7-hydroxy 6,8-dimethoxycoumarin (7H-6,8DM), 7-hydroxy 6-methoxycoumarin (7H-6M), and 7-hydroxy 4-methylcoumarin (7H-4M) in the melanogenic cells model for murine B16F10 melanoma cells. The initial results indicated that melanin production and intracellular tyrosinase activity were significantly stimulated by 7H-4M but not by 7H-5,6DM, 7H-6,8DM, or 7H-6M. Therefore, our present study further investigated the melanogenic effects of 7H-4M in B16-F10 cells, as well as its mechanisms of action. In a concentration-dependent manner, 7H-4M increased intracellular tyrosinase activity, leading to the accumulation of melanin without affecting the viability of B16-F10 cells. Our study further investigated the effects of 7H-4M on melanogenesis, including its ability to promote tyrosinase activity, increase melanin content, and activate molecular signaling pathways. The results indicate that 7H-4M effectively stimulated tyrosinase activity and significantly increased the expression of melanin synthesis-associated proteins, such as microphthalmia-associated transcription factor (MITF), tyrosinase, tyrosinase-related protein-1 (TRP1), and TRP2. Based on our findings, we can conclude that 7H-4M has the ability to activate the melanogenesis process through the upregulation of cAMP-dependent protein kinase (PKA) and the cAMP response element-binding protein (CREB). Additionally, our study showed that 7H-4M induced melanogenic effects by downregulating the extracellular signal-regulated kinase (ERK) and the phosphatidylinositol 3 kinase (PI3K)/protein kinase B (Akt)/glycogen synthesis kinase-3β (GSK-3β) cascades, while upregulating the JNK and p38 signaling pathways. Finally, the potential of using 7H-4M in topical applications was tested through primary human skin irritation tests. During these tests, no adverse reactions were induced by 7H-4M. In summary, our results indicate that 7H-4M regulates melanogenesis through various signaling pathways such as GSK3β/β-catenin, AKT, PKA/CREB, and MAPK. These findings suggest that 7H-4M has the potential to prevent the development of pigmentation diseases.

## 1. Introduction

Melanin is a biological pigment that exists in different parts of the body, such as the skin, hair, eyes, ears, and brain, and plays an important role in preventing skin cancer caused by ultraviolet (UV) light. The overall level of melanin production changes due to aging, stress, and UV damage, resulting in gray hair, sunburn, and spots on the skin. Therefore, controlling melanin production is important for maintaining the health and cosmetic appearance of the human body. Many studies have investigated new pharmacology and herbal formulations aimed at controlling skin pigmentation, with the aim of developing tanning cosmetics to induce beautiful and natural matte tanning, and to treat or prevent hypopigmentation diseases [1,2,3].

Melanin biosynthesis occurs within melanosomes and is mainly regulated by specific enzymes involved in melanogenesis, such as tyrosinase (TYR), tyrosinase-related protein 1 (TRP-1), and TRP-2 (dopachrome tautomerase). TYR plays a critical role as the rate-limiting enzyme in the oxidation reaction of melanin production and is involved in various enzymatic reactions. TRP-2 acts as a dopachrome tautomerase, converting dopachrome to 5,6-dihydroxyl indole-2-carboxylic acid (DHICA), while TRP-1 oxidizes and polymerizes DHICA to form carboxylated indole quinone. The production of these three enzymes is transcriptionally regulated by microphthalmia-associated transcription factor (MITF), which is the master regulator of the differentiation, pigmentation, proliferation, and survival of melanocytes [4]. In addition, pharmacological research has verified that MTF is regulated through various signal pathways, such as the cyclic adenosine monophosphate (cAMP)-mediated pathway, the mitogen-activated protein kinase (MAPK), the phosphatidylinositol 3 kinase (PI3K)/AKT pathway, and the Wnt signaling pathway at a transcriptional or post-translational level. Thus, modulating the activity of enzyme cascades consisting of melanogenic enzymes (i.e., TRY, TRP-1, and TRP-2) and transcription factors is considered an important strategy in the development of skin pigmentation treatments [5].

Coumarin derivatives, which are natural products containing benzopyrone structures, have been widely found as secondary metabolites in plants. These natural coumarins have been shown to exhibit various bioactivities, and have as anti-inflammatory, antitumor, neuroprotective, melanogenic, and anti-melanogenic effects [6,7,8,9]. In view of this, the coumarin derivatives have been widely utilized in the cosmetics, agricultural, and medicine sectors.

**Figure 1:**
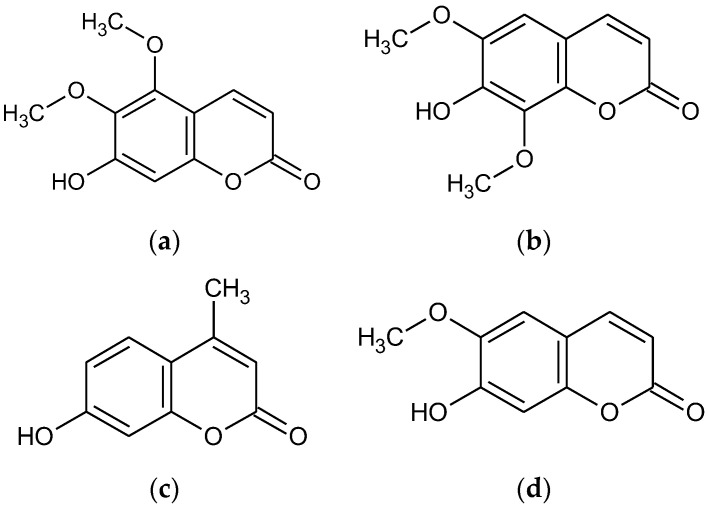
The structure of the 7-hydroxy coumarin derivatives: (**a**) 7-hydroxy-5,6-dimethoxycoumarin, (**b**) 7-hydroxy-6,8-dimethoxycoumarin, (**c**) 7-hydroxy-4-methlycoumarin, and (**d**) 7-hydroxy-6-methoxycoumarin.

As part of our ongoing screening program aimed at discovering new cosmeceuticals and nutraceuticals, we have reported that several coumarin and psoralen derivatives exhibit anti-inflammatory and melanogenic effects [9,10,11,12]. As an extension of this study, we screened four 7-hydroxy coumarin derivatives—7-hydroxy 5,6-dimethoxycoumain (7H-5,6DM), 7-hydroxy 6,8-dimethoxycoumain (7H-6,8DM), 7-hydroxy 6-methoxycoumain (7H-6M), and 7-hydroxy 4-methlycoumarin (7H-4M)—to identify the structural features involved in the melanogenesis of this class of molecules (Figure 1). Several studies have previously focused on this research area, i.e., the functional effects of 7-hydroxy coumarin derivatives on human health. For instance, Veselinović and colleagues assessed the potential anti-melanogenic properties of 7-hydroxy-4-phenylcoumarin and 5,7-dihydroxy-4-phenylcoumarin in zebrafish [13]. In another study, Bhattacharyya and colleagues demonstrated the anti-oncogenic effects of a plant-derived coumarin (7-hydroxy-6-methoxy coumarin) against 7,12-dimethylbenz [a] anthracene-induced skin papilloma in mice, and also found that 4-methyl-7-hydroxy coumarin has anti-cancer potential against DMBA-induced skin cancer in mice [14]. In addition, while research studies have reported the application of 7-hydroxy-coumatin derivatives to vitiligo and their resultant effect of increasing melanogenesis, the four 7-hydroxy-coumatin derivatives in melanogenesis have not been compared and their mechanism of action is unknown, especially for 7H-4M. Thus, the main objective of this study was to investigate the impact of 7H-4M on the molecular mechanism of melanin biosynthesis in B16-F10 mouse melanoma cells. Our findings revealed that the melanogenic properties of 7H-4M were associated with the upregulation of MITF gene expression, which was mediated by the activation of GSK3β/β-catenin, AKT, PKA/CREB, and MAPK signaling pathways. Additionally, we conducted primary skin irritation tests on human subjects to examine the potential use of 7H-4M as a topical agent for inducing melanogenesis.

## 2. Results

### 2.1. The Effect of the 7-Hydroxy Coumarin Derivatives on the Viability of B16-F10 Cells

**Figure 2:**
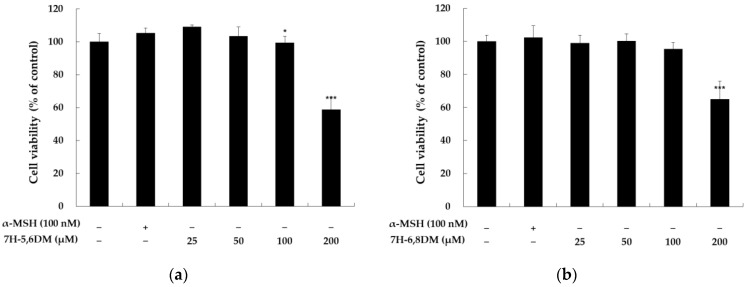

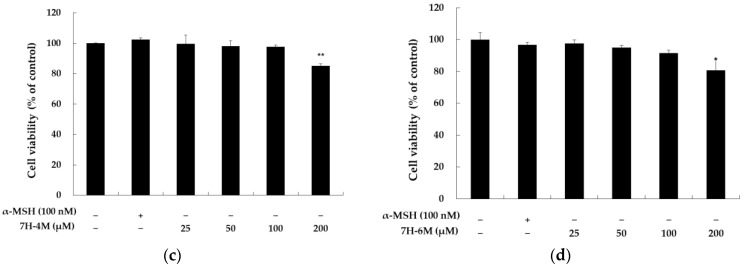
The effect of 7H-5,6DM (**a**), 7H-6,8DM (**b**), 7H-4M (**c**), and 7H-6M (**d**) on the viability of B16-F10 melanoma cells. The cells were plated in 24-well plates (8 × 10^3^ cells/well), incubated for 24 h, and treated with 7H-5,6DM (25, 50, 100, and 200 µM), 7H-6,8DM (25, 50, 100, and 200 µM), 7H-4M (25, 50, 100, and 200 µM), 7H-6M (25, 50, 100, and 200 µM), and α-MSH (100 nM) for 72 h. The cytotoxicity of 7H-5,6DM, 7H-6,8DM, 7H-4M, and 7H-6M was evaluated using MTT assays. The results are presented as the mean ± SD of independent experiments. * *p* < 0.05, ** *p* < 0.01, *** *p* < 0.001 vs. the unstimulated control group.

To investigate whether the 7-hydroxy coumarin derivatives exert cytotoxicity against B16-F10 cells, the cells were treated with various concentrations (25, 50, 100, and 200 μM) of the 7-hydroxy coumarin derivatives and α-MSH (100 nM) for 72 h. There were no significant differences for up to 100 μM of the 7-hydroxy coumarin derivatives in the B16-F10 cells (Figure 2). The cell viability of 7H-5,6DM, 7H-6,8DM, 7H-6M, and 7H-4M was reduced by 99.5, 95.4, 97.7, and 91.4%, respectively, compared to the control group (cell viability decline rate below 20%) [15]. Therefore, we used the 7-hydroxy coumarin derivatives at concentrations below 100 μM for further experiments.

### 2.2. The Effect of the 7-Hydroxy Coumarin Derivatives on the Melanin Content and Tyrosinase Activity of B16-F10 Cells

**Figure 3:**
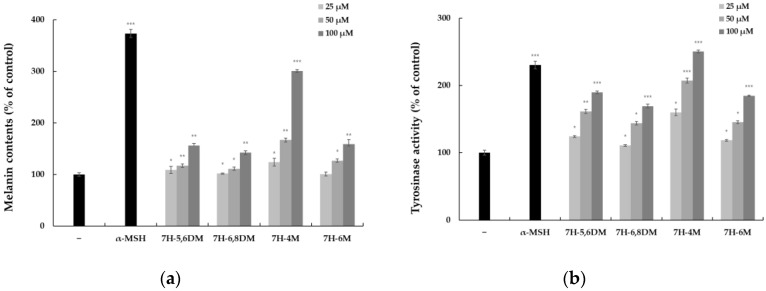
The effect of 7H-5,6DM, 7H-6,8DM, 7H-4M, and 7H-6M on the production of melanin (**a**) and tyrosinase activity (**b**) in B16-F10 melanoma cells. The cells were plated in 6-well plates (5 × 10^4^ cells/well), incubated for 24 h, and treated with 7H-5,6DM (25, 50, and 100 μM), 7H-6,8DM (25, 50, and 100 μM), 7H-4M (25, 50, and 100 μM), 7H-6M (25, 50, and 100 μM), and α-MSH (100 nM) for 72 h. α-MSH was used as the negative control. The results are presented as the mean ± SD from independent experiments. * *p* < 0.05, ** *p* < 0.01, *** *p* < 0.001 vs. the control group.

To examine whether the 7-hydroxy coumarin derivatives affected the melanogenesis of B16-F10 cells, the cells were treated with the 7-hydroxy coumarin derivatives (25, 50, and 100 μM) and α-MSH (100 nM) for 72 h. The intracellular melanin accumulation and tyrosinase activity increased significantly in the B16-F10 cells. We used α-MSH as a positive control. As shown in Figure 3, 7H-5,6DM, 7H-6,8DM, 7H-6M, and 7H-4M also stimulated the accumulation of melanin and tyrosinase activity in the B16-F10 cells in a concentration-dependent manner. Among them, 7H-4M enhanced the melanin content and tyrosinase activity more than other 7-hydroxy coumarin derivatives, and the rate of melanin accumulation and tyrosinase activity in cells at a concentration of 100 μM was 2.5 times higher than the untreated control group. B16-F10 cells treated with 7H-4M showed no significant difference compared to the α-MSH (100 nM)-treated group. As a result, we conducted additional experiments to assess the melanogenic effects of 7H-4M.

### 2.3. The Effect of 7H-4M on the Expression of Melanogenic Enzymes and MITF

**Figure 4:**
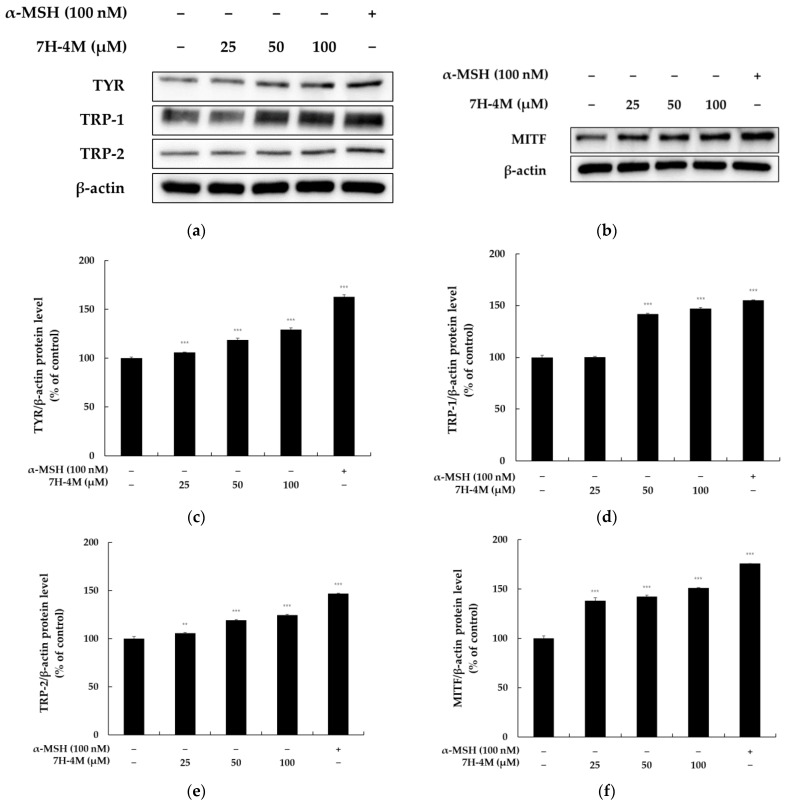
The effect of 7H-4M on the melanogenesis-related protein expression of B16-F10 cells. The cells were treated with various concentrations of 7H-4M for 72 h and were collected for protein extraction. The expression levels of tyrosinase (TYR), TRP-1, TRP-2, and MITF were evaluated using a Western blot experiment. (**a**,**b**) Western blotting results and (**c**) tyrosinase, (**d**) TRP-1, (**e**) TRP-2 and (**f**) MITF protein expression. The protein bands were quantified using ImageJ software, and the intensity values were normalized to the loading control. The untreated cells were considered as 100%, and the values obtained from treated cells were compared to the control group. The results are presented as mean ± SD of at least three independent experiments. Statistical analysis showed that there were significant differences between the control group and the 7H-4M-treated groups at concentrations of 25, 50, and 100 μM for TYR, TRP-1, TRP-2, and MITF. The significance levels were *** *p* < 0.001 and ** *p* < 0.01.

To investigate the impact of 7H-4M on the expression of melanogenic enzymes such as tyrosinase, TRP-1, and TRP-2 and their transcriptional regulator MITF, a Western blot analysis was conducted. The results depicted in Figure 4 revealed a significant increase in the protein expression of tyrosinase, TRP-1, and TRP-2 at concentrations of 25, 50, and 100 μM of 7H-4M, compared to the untreated control group. Furthermore, 7H-4M also upregulated the expression of MITF. All protein expression levels showed 7H-4M concentration-dependent effects. These results show that 7H-4M enhanced melanogenesis enzymes, without eliciting cytotoxic effects, by increasing MITF expression and melanogenesis.

### 2.4. The Effect of 7H-4M on the Wnt/β-Catenin Signaling Pathway

**Figure 5:**
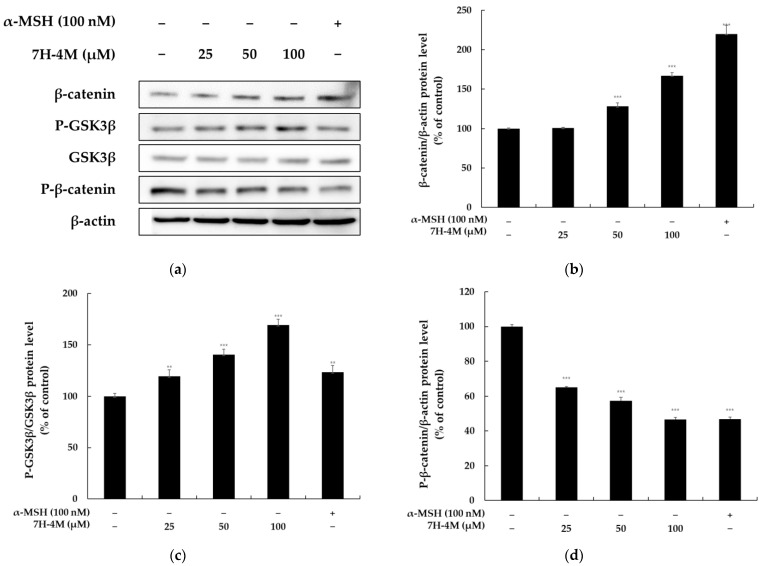
The effect of 7H-4M on the melanogenesis-related protein expression of B16-F10 cells. The experimental protocol involved treating B16-F10 cells with 7H-4M at varying concentrations for 24 h. The cells were then collected and protein extracts were prepared from each group. The expression levels of P-GSK3β, P-β-catenin, and β-catenin were assessed through Western blot analysis. The relative intensity of the protein bands was measured using ImageJ software, and the values were normalized to the corresponding loading control. The untreated cells were considered as having 100% expression. (**a**) Western blotting results and (**b**) β-catenin, (**c**) P-GSK3, and (**d**) P-β-catenin protein expression. The reported values are the mean ± SD of at least three independent experiments, and the significance level is shown as ** *p* < 0.01 and *** *p* < 0.001 compared to the control group.

It has been reported that the GSK-3β/β-catenin pathway is involved in melanogenesis via the regulation of MITF and TYR expression. Specifically, GSK-3β leads to the accumulation of β-catenin in the cytoplasm and eventual nuclear translocation, finally resulting in the transcriptional activation of MITF, following the expression of melanogenic enzymes [9,16]. In this study, we aimed to determine whether 7H-4M induces melanogenesis in B16-F10 cells via the Wnt/β-catenin signaling pathway. To investigate this, we performed experiments and found that 7H-4M increased the expression of P-GSK3β and β-catenin compared to the untreated group, as shown in Figure 5. However, 7H-4M inhibited the expression of P-β-catenin compared to the untreated group. These results suggest that 7H-4M induces melanogenesis through the Wnt/β-catenin signaling pathway.

### 2.5. The Effect of 7H-4M on the AKT Signaling Pathway

**Figure 6:**
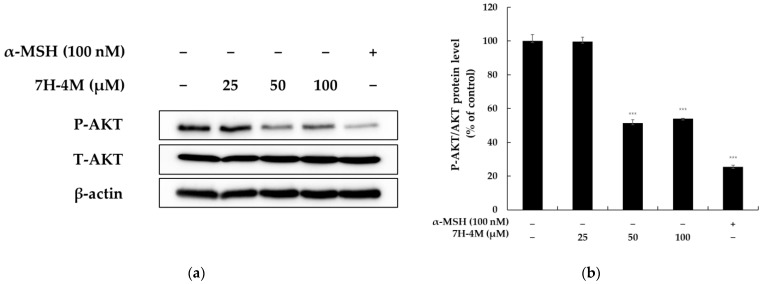
The effect of 7H-4M on the melanogenesis-related protein expression of B16-F10 cells. The cells were treated with 7H-4M at the indicated concentrations for 24 h and were then harvested. Protein extracts were prepared from each treatment group to evaluate the expression of P-AKT. The relative intensity of the protein band was quantified using ImageJ software, and the value was normalized to that of the corresponding loading control. The untreated cells were considered as 100%. (**a**) Western blotting results and (**b**) P-AKT protein expression. The values are presented as the mean ± SD of at least three independent experiments. Statistical analysis was performed using *** *p* < 0.001 compared to the control group.

It has been reported that the activation of the protein kinase AKT mediates the phosphorylation of GSK-3β, which induces melanin synthesis via the upregulation of MITF. In other words, blocking the phosphorylation of AKT triggers the process of melanogenesis [17]. To investigate whether 7H-4M induces melanogenesis through the AKT signaling pathway in B16-F10 cells, we conducted experiments. The results showed that 7H-4M reduced the phosphorylation of AKT (Figure 6). Thus, these findings suggest that 7H-4M induces melanogenesis by inhibiting AKT phosphorylation in the AKT signaling pathway.

### 2.6. The Effect of 7H-4M on the PKA/CREB Signaling Pathway

**Figure 7:**
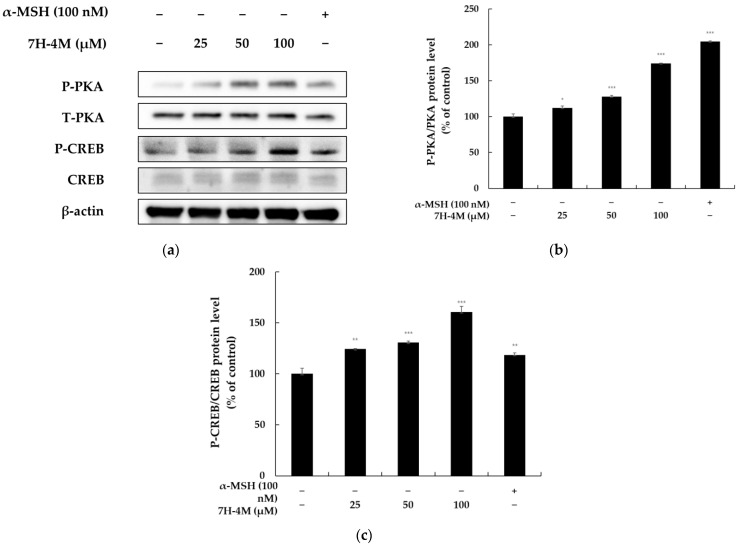
The effect of 7H-4M on the melanogenesis-related protein expression of B16-F10 cells. The cells were treated with 7H-4M at specified concentrations for 24 h, followed by harvesting. Protein extracts were then prepared from each treatment group to evaluate the expressions of P-PKA and P-CREB. Using ImageJ software, the relative intensity of the protein band was quantified and normalized to that of the corresponding loading control. The untreated cells were considered as 100%. (**a**) Western blotting results and (**b**) P-PKA, and (**c**) P-CREB protein expression. The results are shown as mean ± SD of at least three independent experiments, with *** *p* < 0.001, ** *p* < 0.01, and * *p* < 0.05 indicating significance compared to the control group.

In previous studies, it was found that the cAMP response element-binding protein (CREB), which is phosphorylated by protein kinase A (PKA), activates melanogenesis-related proteins, promotes MITF transcription, and induces melanin synthesis. Therefore, we investigated the effects of 7H-4M on the phosphorylation and/or expression of PKA/CREB, potential upstream mediators, to determine their involvement in melanogenesis. We examined whether 7H-4M induces melanogenesis through the PKA signaling pathway in B16-F10 cells. The results showed that 7H-4M increased the phosphorylation of PKA and CREB compared to the untreated group (Figure 7). These findings suggest that 7H-4M promotes melanogenesis through the PKA/CREB signaling pathway.

### 2.7. The Effect of 7H-4M on the MAPK Signaling Pathway

**Figure 8:**
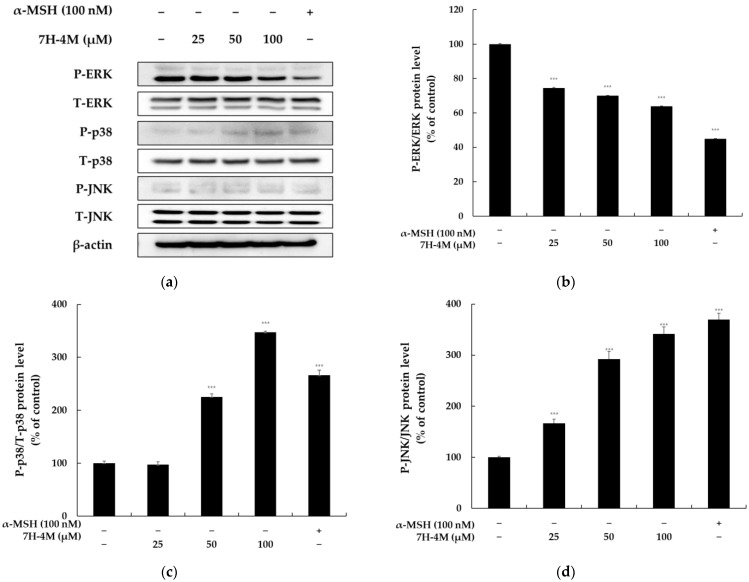
The effect of 7H-4M on the melanogenesis-related protein expression of B16-F10 cells. The experiment involved treating cells with various concentrations of 7H-4M for 24 h, followed by harvesting the cells. Protein extracts were prepared from each treatment group and the expressions of MAPKs (P-ERK, P-p38, and P-JNK) were evaluated. The relative intensity of the protein band was quantified using ImageJ software and the value was normalized to that of the corresponding loading control. Untreated cells were used as the control group and were considered to have a value of 100%. (**a**) Western blotting results and (**b**) P-ERK, (**c**) P-p38, and (**d**) P-JNK protein expression. The results were shown as the mean ± SD of at least three independent experiments, with statistical significance indicated by *** *p* < 0.001.

To further confirm the pathways involved in the melanogenic effects of 7H-4M, we analyzed MAPK levels by Western blotting. As shown in Figure 8, the levels of P-p38 and P-JNK increased markedly in response to 100 μM of 7H-4M compared to the levels in B16-F10 cells; the phosphorylation of ERK also markedly decreased in a concentration-dependent manner. These results were consistent with the substances having melanogenic effects, suggesting that the MAPK signaling pathway was involved in the melanogenic effect of 7H-4M [9].

### 2.8. Effects of 7H-4M on Signaling Pathways by Specific Inhibitors

**Figure 9:**
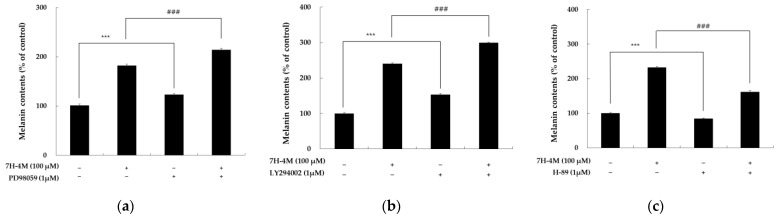
Effect of 7H-4M on production of melanin in a B16F10 melanoma cells. To evaluate the role of ERK, AKT, and PKA in melanin synthesis induced by 7H-4M, B16F10 cells were co-treated with specific inhibitors PD98059 (ERK inhibitor, 1 μM), LY294002 (AKT inhibitor, 1 μM), and H-89 (PKA inhibitor, 1 μM) for 72 h. Melanin content was measured and compared to untreated cells, which were regarded as 100%. The results showed that PD98059 (**a**), LY294002 (**b**), and H-89 (**c**) significantly decreased melanin content induced by 7H-4M. Values are shown as the mean ± SD of at least three independent experiments. *** *p* < 0.001 compared to the control group. ### *p* < 0.001 compared to the 7H-4M group without specific inhibitor.

We used specific inhibitors, including ERK inhibitor (PD98059), PKA inhibitor (H-89), and AKT inhibitor (LY294002), to confirm the role of signal pathways in 7H-4M induced melanin synthesis. One hour before adding 7H-4M, we pretreated cells with these inhibitors and incubated them for 72 h to measure melanin content. As depicted in Figure 9, 7H-4M treatment increased melanin content, and this effect was significantly enhanced by PD98059 and LY294002. However, when we treated cells with H-89 and 7H-4M simultaneously, the melanin content only slightly increased compared to 7H-4M alone. These findings suggest that ERK–PKA–AKT-mediated signaling is crucial for increasing melanin production induced by 7H-4M.

### 2.9. Human Primary Irritation Test of 7H-4M

We conducted primary human skin irritation tests to assess the potential of 7H-4M for topical application. We applied acenocoumarol to patches of skin at concentrations of 50 and 100 µM for up to 24 h. We then observed the patches 20 min and 24 h after removing the 7H-4M. Squalene (solvent) was used as a negative control. According to the results presented in Table 1, the test substance (7H-4M) was classified as causing “no to slight irritation” in terms of its primary irritation potential on human skin.

## 3. Discussion

Coumarins are a type of organic compound that have a fused benzene and α-pyrone ring structure, belonging to the larger subclass of benzopyrones. They are commonly found in various dietary plants, particularly in cinnamon and many fruits and vegetables [18]. Different derivatives of these compounds exhibit diverse biological activities, including antioxidant, anti-inflammatory, antimicrobial, and in vitro and in vivo platelet anti-aggregation effects [10,11,18,19,20,21]. Natural coumarins and their derivatives have always provided an invaluable source for the discovery of novel melanogenic and/or tanning agents. Natural coumarins, such as imperatorin, 7,8-dimethoxycoumarin, and psoralen derivatives, as well as several coumarin-containing plant extracts, have been reported to activate the melanogenesis process in B16-F10 cell lines [8,9,10,11].

From the use of our screening strategy for multi-targeting agents, our laboratory re-ported attractive results that elucidate the functionalities of various psoralens and coumarins in terms of their anti-inflammatory and melanogenesis-stimulating effects [8,9,10,11,12,13]. In this study, we aimed to verify the efficacy of the 7-hydroxy coumarin derivatives and systematically applied this to skin-related diseases using four 7-hydroxy coumarin derivatives: 7-hydroxy 5,6-dimethoxycoumain (7H-5,6DM), 7-hydroxy 6,8-dimethoxycoumain (7H-6,8DM), 7-hydroxy 6-methoxycoumain (7H-6M), and 7-hydroxy 4-methlycoumarin (7H-4M) (Figure 1). In the present study, we showed the promoting effects of the 7-hydroxy coumarin derivatives on melanogenesis based on their capacity to inhibit the melanin content and cellular tyrosinase activity. In addition, 7H-4M exhibited more stimulatory effects on melanin production in mouse B16-F10 melanoma cells than other 7-hydroxy coumarin derivatives (Figure 2). Previous studies have shown that 7H-4M and its derivatives possess several biological properties. Known as a potent xanthine oxidase inhibitor, 7-hydroxy 4-methylcoumarin is effective in inhibiting both the settlement as well as the byssogenesis of mussels, modulating some of the effector functions of human neutrophils and exhibiting antioxidant activity [22,23,24,25]. We previously reported that 7H-4M alleviates the LPS-induced inflammatory response in RAW264.7 macrophage cells [26]. Furthermore, we also observed that 7,8-diacetoxy-4-methylcoumarin and 7,8-diacetoxy-4-methylthiocoumarin possess an in vitro anticancer effect. However, extensive studies on melanogenesis and the structure–cytotoxic activity of the 7-hydroxy coumarin derivatives have not yet been performed. In the present study, we showed the promoting effects of the 7-hydroxy coumarin derivatives on melanogenesis based on their capacity to increase the melanin content and cellular tyrosinase activity. In addition, 7H-4MC exhibited more stimulatory effects on melanin production in mouse B16-F10 melanoma cells than other 7-hydroxy coumarin derivatives (Figure 3). In terms of the structure–activity relationship, it may be assumed that replacement of the methoxy group in 7-hydroxy coumarin’s C-5, C-6, and C-8 positions, and the methyl group in the C-6 position, resulted in a moderate melanogenesis enhancement effect, while the replacement of the methyl group in the C-4 position resulted in a dramatic increase in melanogenesis. Interesting studies have been reported on the additional 4-methylcoumarin structure of 7H-4M. One of the favorable properties of 4-methylcoumarins is that they are less likely to be metabolized to the mutagenic derivative 3,4-coumarin epoxide by the liver cytochrome P450 enzyme due to the presence of a methyl group at the C4 position. In addition, 4-methylcumarin is not presumed to have the anti-coagulant effect of warfarin derivatives, and the presence of the OH portion in C4 is an important feature of its efficacy [27]. Finally, although there have been many reports that the methoxylation of coumarin or flavonoid structures activates melanogenesis, our study is the first to show that methylation activates melanogenesis. In general, structures with many hydroxyl groups have high free radical scavenging capacity, and this antioxidant capacity is associated with melanogenesis inhibitory activity, so it is predictable that methoxyl group substitution in the 7-hydroxy coumarin structure could activate melanogenesis. The fact that methylation of the C-4 position is significantly more melanogenic than methoxylated coumarin is an interesting finding, and it will be necessary to continue to investigate whether the absorbance or the transmission plays a role in UVreflection or in triggering certain melanocyte activation pathways.

Melanogenesis is a complex mechanism that is related to at least 120 to 130 genetic loci. Among the genes involved in melanogenesis, the tyrosinase family, which consists of tyrosinase, tyrosinase-related protein-1 (TRP-1), and TRP-2, has been recognized as containing the most critical regulators of melanin biosynthesis. As expected, our results showed that the 7H-4M concentration increased melanin production via the induction of the melanogenic enzyme tyrosinase and the pigmentation-related transcription factor MITF in the B16-F10 model system (Figure 4). These data suggest that one of the mechanisms for 7H-4M-induced melanogenesis in B16-F10 cells is associated with the increase in the expression of the melanogenic enzyme tyrosinase through a master regulator of melanocyte activity, MITF. Interestingly, these results are inconsistent with those of previous studies, which reported that 7-hydroxy-4-phenylcoumarin has an anti-melanogenesis effect [13]. We believe that the conflicting results obtained for the methyl group and phenyl group substitution on the 7-hydroxy coumarin C-4 position could be an interesting strategy for the selective discovery of melanogenesis regulators through organic synthesis in the future.

Several signal transduction pathways that are involved in melanogenesis regulate MITF and TYR expression, including the GSK-3β/Wnt/β-catenin, the PI3K/AKT, the PKA/CREB, and the mitogen-activated protein kinase (MAPK) signaling pathways [28].

The Wnt/β-catenin signaling pathway involves the GSK-3β and β-catenin proteins. GSK-3β is a kinase that is constantly active and can be phosphorylated by several other kinases, including AKT and PKA. Recent research has shown that the β-catenin pathway is closely related to melanogenesis; as β-catenin moves from the cytoplasm to the nucleus, it promotes MITF transcription, upregulates MITF expression, and then binds to lymphocyte-enhancing factor (LEF) [16,29]. In this study, we observed that 7H-4M induced the phosphorylation of GSK-3β, resulting in the accumulation of β-catenin in the cytoplasm. Our results also demonstrated that 7H-4M promoted the accumulation of β-catenin in the cytoplasm, which led to the overexpression of MITF and ultimately stimulated melanin biosynthesis (Figure 5). Therefore, the production of melanin by 7H-4M was likely induced via the Wnt pathway, consistent with reports indicating that the activation of GSK3/β-catenin affects melanin production in B16-F10 melanoma cells.

Previous studies have shown that the PI3K/AKT signaling pathway is another important pathway that regulates the transcriptional activity of MITF. The activation of PI3K/AKT has been shown to inhibit melanin accumulation in both murine melanocytes and human melanoma melanin A cells [30,31]. Therefore, it is important to ascertain whether 7H-4M activates or inhibits the PI3K/AKT pathway. As expected, our data showed that the P-AKT levels were downregulated in 7H-4M-induced melanogenesis in B16-F10 cells, leading to the increased expression of tyrosinase, TRP-1, and MITF (Figure 6). This suggests that the PI3K/AKT pathway may contribute to the stimulation of melanogenesis by 7H-4M.

The PKA/CREB signaling pathway is widely recognized as being involved in melanin synthesis. Elevated cellular cAMP can activate PKA, which, in turn, promotes MITF transcription activity through phosphorylation of CREB. This leads to the increased expression of proteins such as tyrosinase, TRP-1, and TRP-2, ultimately resulting in melanin production [32,33]. To investigate whether the PKA/CREB signaling pathway mediates the effect of 7H-4MC on melanogenesis, Western blot experiments were performed to evaluate the enhancement of PKA and CREB phosphorylation. The results depicted in Figure 7 showed that 7H-4M-induced melanin synthesis strongly increased PKA and CREB phosphorylation in a concentration-dependent manner, indicating the involvement of the PKA/CREB signaling pathway in 7H-4M-induced melanogenesis.

Furthermore, the MAPK family, including ERK, JNK, and p38 MAPK, plays an important role in melanogenesis. Studies have shown that p38 MAPK is an important intracellular signaling molecule for pigmentation. ERK and JNK are also involved in regulating melanogenesis [34,35]. Several studies have shown that the overall role of MAPK pathway activation in melanin production is controversial. In B16-F10 cells, Schisandrin B inhibited melanin production by decreasing the phosphorylation levels of ERK [36]. In contrast, two studies have proven that increased P-ERK levels suppress melanogenesis [8,9]. These conflicting results, due to the complexity of melanin synthesis regulation, are often explained in relation to the process of inducing the ubiquitin proteasome-dependent degradation of MITF. Therefore, we investigated whether 7H-4M induces JNK and p38 MAPK or ERK inhibition in B16-F10 cells. Figure 8 shows that the 7H-4M treatment significantly increased the phosphorylation of JNK and p38 MAPK at 4 h, while the phosphorylation level of ERK decreased. These results suggest that the 7H-4M treatment stimulated pigmentation by functionally regulating JNK and p38 MAPK activation and inhibiting ERK signaling.

This is the first study to demonstrate 7H-4M-related melanin induction and its molecular mechanism. Our results suggest that activating MITF through the PI3K/AKT, GSK3β/β-catenin, PKA/CREB, and MAPK pathways mediates the hypopigmentary effect of 7H-4M. Accordingly, we propose that 7H-4M may be used as a potential therapeutic agent to treat hypopigmentation and as a useful ingredient for human health. Our study has some limitations that need to be addressed. Although 7H-4M exhibited a melanogenic effect at the cellular level, it does not necessarily translate to similar outcomes in human melanocytes or clinical studies. Thus, to apply the results practically, additional studies on human melanocytes or clinical trials are required to establish the therapeutic regimen of 7H-4M for the treatment of hypopigmentary disorders in humans.

## 4. Materials and Methods

### 4.1. Materials

The 7-hydroxy 5,6-dimethoxycoumain (7H-5,6DM; purity 98%), 7-hydroxy 6,8-dimethoxycoumain (7H-6,8DM; purity 98%), and 7-hydroxy 4-methylcoumarin (7H-4M; purity 98%) used in this study were purchased from ChemFaces (Wuhan, Hubei, China). The 7-hydroxy 6-methoxycoumain (7H-6M; purity 99%) was purchased from Sigma-Aldrich Co. (St. Louis, MO, USA). The α−melanocyte-stimulating hormone (α−MSH), protease/phosphatase inhibitor cocktail, sodium hydroxide (NaOH), arbutin, and L−DOPA, were obtained from Sigma-Aldrich Co. (St. Louis, MO, USA). Dulbecco’s modified Eagle’s medium (DMEM), penicillin−streptomycin, and 0.5% trypsin−ethylenediaminetetraacetic acid (10×) were purchased from Thermo Fisher Scientific (Waltham, MA, USA), and the fetal bovine serum (FBS) was obtained from Merck Millipore (Burlington, MA, USA). The 3−(4,5−dimethylthiazol−2−yl)−2,5−diphenyltetrazolium bromide (MTT), dimethyl sulfoxide (DMSO), phosphate-buffered saline (PBS), tris-buffered saline (TBS), sodium dodecyl sulfate (SDS), radioimmunoprecipitation assay (RIPA) buffer, and enhanced chemiluminescence (ECL) kit were all purchased from Biosesang (Seongnam, Gyeonggi-do, Republic of Korea). The BCA protein assay kit was purchased from Thermo Fisher Scientific (Waltham, MA, USA), and the Tween 20 and 2× Laemmli sample buffer was obtained from Bio-rad (Hercules, CA, USA). The skim milk was purchased from BD Difco (Franklin Lakes, NJ, USA). The primary antibodies tyrosinase, TRP-1, TRP-2, and MITF, which were used for the Western blot, were purchased from Santa Cruz Biotechnology (Dallas, TX, USA), and phospho-specific P-ERK (Thr202/Tyr204, #9101S), ERK, phospho-specific P-p38 (Thr180/Tyr182, #9211S), p38, phospho-specific P-JNK (Thr183/Tyr185, #9251S), JNK, phospho-specific P-PKA (Thr197, #5661S), PKA, phospho-specific P-AKT(Ser473, #9271S), AKT, phospho-specific P-GSK−3β (Ser9, #9322S), GSK−3β, phospho-specific P-β−catenin (Ser33/37/Thr41, #9261S), β−catenin, β−actin, and secondary antibodies anti-mouse and anti-rabbit were purchased from Cell Signaling Technology (Danvers, MA, USA). For the inhibitors used in the study, PD98059 (ERK inhibitor), LY294002 (AKT inhibitor), and H-89 (PKA inhibitor) were purchased from Cayman Chemicals, Cell Signaling, and Sigma-Aldrich, respectively.

### 4.2. Cell Culture

The B16-F10 cell line was isolated from the skin tissue of a mouse with melanoma that produced large amounts of melanin. The B16-F10 cells were incubated in DMEM supplemented with 10% FBS and 1% penicillin/streptomycin at 37 °C in a humidified, CO_2_-controlled (5%) incubator (NB-203XL, N-BIOTEK, Inc., Bucheon, Republic of Korea).

### 4.3. MTT Assay

The general viability of the cultured cells was determined through the reduction of MTT to formazan. The B16-F10 cells (8 × 10^3^ cells/well) were seeded on 24-well plates. After treatment with 7-hydroxy coumarins, the cells were incubated for 72 h at 37 °C in a 5% CO_2_ atmosphere. MTT (5 mg/mL) was added to each well at a 1/10 volume of the medium. The cells were incubated at 37 °C for 4 h, and DMSO was added in order to dissolve the formazan crystals. The absorbance was then measured at 570 nm using a spectrophotometer (Epoch, BioTek Instruments, Charlotte, VT, USA).

### 4.4. Measurement of the Melanin Content

The melanin content was measured as described previously, with a slight modification [8,9,10]. Briefly, the cells were seeded in 6-well plates at a density of 5 × 10^4^ cells and were allowed to attach overnight. Following this, the cells were incubated with various concentrations of 7-hydroxy coumarins for 3 days. The cells were then washed twice with ice-cold PBS, lysed with RIPA buffer, and centrifuged at 15,000 rpm for 20 min. The supernatants were analyzed for protein concentration; the pellets were dissolved at 80 °C for 10 min by treating 200 μL of 1 N NaOH. The optical densities (OD) of the supernatants were measured at 405 nm using an ELISA reader (Epoch, BioTek Instruments, Charlotte, VT, USA).

### 4.5. Measurement of Tyrosinase Activity

The tyrosinase activity was determined as previously described with slight modifications [8,9,10]. Briefly, B16-F10 cells were cultured in 6-well plates. The cells were treated with test substances in DMEM for 3 days. After that, the cells were washed with ice-cold PBS and lysed using phosphate buffer (pH 6.8) with 1% Triton X-100. The cells were disrupted by freeze-thawing, and the lysates were clarified by centrifugation at 15,000 rpm for 20 min. After quantifying the protein levels and adjusting the concentrations with a lysis buffer, 20 μL of each lysate was placed in a well of a 96-well plate, and 80 μL of 2 mg/mL L-DOPA was added. The control wells contained 20 μL of lysis buffer and 80 μL of 2 mg/mL L-DOPA. After incubation at 37 °C, absorbance was measured using an ELISA reader (Epoch, BioTek Instruments, Charlotte, VT, USA). The value of each measurement was expressed as the percentage change from the control.

### 4.6. Western Blot Analysis

The B16-F10 cells were seeded in a 60 mm cell culture dish at 7 × 10^4^ cells for 24 h. The cells were treated with 7-hydroxy 4-methycoumarin (25, 50 and 100 μM) for each protein expression time. After incubation, they were washed with 1 × PBS buffer and lysed using RIPA buffer at 4 °C for 20 min. The proteins were separated by 10% sodium dodecyl sulfate-polyacrylamide gel (SDS-PAGE) and transferred to a polyvinylidene fluoride (PVDF) membrane. The membranes were blocked with 5% skim milk for 1 h and washed with 1 × TBS-T for 10 min a total of six times, after which they were probed with the appropriate primary antibodies in a 1:2000 dilution overnight. After washing six times with 1 × TBS-T, the membranes were incubated with a secondary antibody (1:1000) for 2 h, and the bands were visualized using an enhanced chemiluminescence (ECL) system (Biosesang, Seongnam, Republic of Korea).

### 4.7. Primary Skin Irritation Test

For this study, we enrolled 31 healthy female volunteers aged between 20 and 60 years who had never experienced irritant and/or allergic contact dermatitis. The participants’ ages ranged from 38 to 55 years, and their mean age was 47.35 ± 4.34 years. We prepared a negative control, squalene-added 7-hydroxy 4-methycoumarin, and applied it at concentrations of 50 and 100 μM. We followed the PCPC guidelines to assess the primary skin irritation responses and used the formula shown below to calculate the reaction results for each test substance. After obtaining written consent from each volunteer, we conducted the study in accordance with the Helsinki Declaration, as a declaration of the ethical principles for medical research, and the Industrial Review Board (IRB) of Dermapro Inc. (IRB number: 1-220777-A-N-01-B-DICN23003) approved it.

$$\mathrm{Response}=\frac{\sum \left(Grade\times No.of\;Responders\right)}{4\;(Maximum\;Grade)\times n\;(Total\;Subjects)}\times 100\times 1/2$$

### 4.8. Statistical Analyses

The results of the experiments are expressed as the mean and standard deviation (mean ± SD) of three repeated experiments. Statistical significance was determined based on the *p*-values using Student’s *t*-test: # *p* < 0.001 vs. the unstimulated control group; * *p* < 0.05, ** *p* < 0.01, *** *p* < 0.001 vs the control group.

## 5. Conclusions

**Figure 10:**
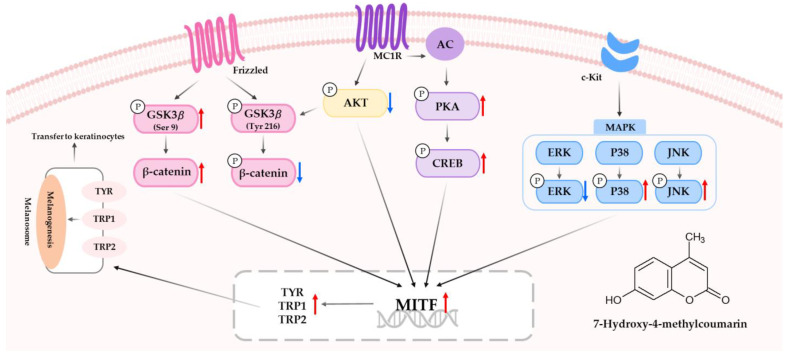
Schematic diagram of the proposed mechanism regulating action of 7H-4M on melanogenesis.

Methoxylated coumarins and flavonoids have been reported to activate melanogenesis, but our study is the first to show that methylated coumarins may also be involved in melanogenesis. Furthermore, we have shown for the first time that the methylated coumarin 7H-4M has better melanogenic activity than the conventional methoxylated coumarins, including the 7-hydroxy coumarin derivatives 7H-5,6DM, 7H-6,8DM, and 7H-6M. After collecting all of the information regarding the melanogenic effects of 7H-4M identified in this study, a map of the relevant molecular pathways was constructed (Figure 10). Results showed that 7H-4M treatment induced melanin synthesis and TYR in B16F10 melanoma cells. 7H-4M was shown to increase melanin synthesis by inducing the expression of melanogenic proteins and transcriptional activity of MITF. Our findings indicate that 7H-4M activates the melanogenesis process by upregulating PKA/CREB, ERK and the PI3K/Akt/GSK-3β cascades, while upregulating the JNK and p38 signaling pathways. We tested the potential of 7H-4M for topical applications by conducting primary human skin irritation tests, which did not induce any adverse reactions. Therefore, 7H-4M is suggested to be a potential melanogenic natural occurring coumarin, and it might be utilized as a cosmetic ingredient against hypopigmentation. However, further studies are needed to fully understand the mechanisms involved in the melanogenic efficacy of 7H-4M using normal melanocytes and in vivo.

## Figures and Tables

**Table 1:** The results from the primary human skin irritation tests (*n* = 31).

No.	Test Sample	No. of Respondents	20 min after Removal				24 h after Removal				Reaction Grade (R) *		
			+1	+2	+3	+4	+1	+2	+3	+4	24 h	48 h	Mean
1	7H-4M (50 μM)	0	-	-	-	-	-	-	-	-	0	0	0
2	7H-4M (100 μM)	0	-	-	-	-	-	-	-	-	0	0	0
3	Squalene	0	-	-	-	-	-	-	-	-	0	0	0

The reactions were assessed 20 min and 24 h after the removal of the treatment by the investigator, according to PCPC guidelines (2014). * The range of irritation from “no to slight irritation”: 0.00 ≤ R < 0.87.

## Data Availability

Not applicable.

## Sample Availability

Samples of the compounds are not available from the authors.
